# Role of Quinoa (*Chenopodium quinoa* Willd) and Chickpea (*Cicer arietinum* L.) Ratio in Physicochemical Stability and Microbiological Quality of Fermented Plant-Based Beverages during Storage

**DOI:** 10.3390/foods13152462

**Published:** 2024-08-04

**Authors:** John Hurtado-Murillo, Wendy Franco, Ingrid Contardo

**Affiliations:** 1 Department of Chemical Engineering and Bioprocesses, Pontificia Universidad Católica de Chile, Ave. Vicuña Mackenna 4860, Santiago 7820436, Chile; jdhurtado@uc.cl; 2 Biopolymer Research & Engineering Laboratory (BiopREL), School of Nutrition and Dietetics, Faculty of Medicine, Universidad de los Andes, Chile, Monseñor Álvaro del Portillo 12.455, Las Condes 7550000, Chile; 3 Centro de Investigación e Innovación Biomédica (CIIB), Universidad de los Andes, Chile, Monseñor Álvaro del Portillo 12.455, Las Condes 7620086, Chile

**Keywords:** quinoa-to-chickpea ratio, physicochemical stability, microbiological quality, storage, plant-based beverages

## Abstract

Three different fermented plant-based beverages were prepared and stored for a long period (50 days) to assess the effect of the quinoa-to-chickpea ratio on physicochemical stability and microbiological quality. Physicochemical stability was evaluated based on pH, acidity, Brix degrees, water-holding capacity (WHC), viscosity, and viscoelasticity. At the end of the long-term storage period, the pH, acidity, and WHC remained stable. During the entire storage period, the beverages maintained good bacterial, fungal, and lactic acid bacteria (LAB) counts. Quinoa and chickpea flour ratios of 50% showed a higher viscosity (18 Pa.s) and WHC (65%) during short-term storage (0–30 d), indicating that the presence of chickpea flour had a positive effect on these parameters, possibly because chickpea starch contains higher amounts of amylose and long-branch chain amylopectin, which impacts the retrogradation pattern under acidic and refrigerated conditions. However, at the end of storage (50 days), the same blend had a higher acidity, lower viscosity (0.78 Pa.s), and lower LAB counts (~1 × 10^8^ CFU/mL), indicating that the increase in chickpea flour had an adverse long-term effect on these parameters. These results suggest that although different ratios of plant sources can improve the physical aspects, they need to be incorporated in a balanced manner to avoid negative effects on both short- and long-term storage, owing to the incorporation of different types of starches and proteins affecting the stability of the system.

## 1. Introduction

In recent years, the production and consumption of plant-based beverages (PBBs) have increased exponentially as consumers explore new food options. The global PBB market is estimated to be worth USD 12.1 to USD 18.5 billion by 2022, and it is projected to reach over USD 24 billion by 2025 [1]. PBBs have gained popularity as a complement or substitute for dairy products, particularly among people who are lactose-intolerant, those who are allergic to cow’s milk protein, and those who follow a vegan, vegetarian, or flexitarian lifestyle [2]. Also, there are growing ethical issues related to animal welfare, the negative environmental impacts associated with dairy production, and the rising process of animal-derived food. PBBs are aqueous extracts obtained through the breakdown (size reduction) and homogenization of the raw materials. From a physicochemical perspective, PBBs are colloidal suspensions or emulsions of dispersed plant materials, where oil is the dispersed phase and water is the aqueous/continuous phase [2,3]. The predominant sources of PBBs are cereals (rice, wheat, and barley), legumes (soybean, bean, and cowpeas), nuts (almonds and peanuts), seeds (pumpkin, hemp, and sunflower seeds), pseudo-cereals (quinoa, amaranth, and buckwheat), and coconuts. However, the production of PBBs faces technological challenges during cold storage due to their low physicochemical stability (phase separation) and microbiological quality related to improper cleaning and poor sanitation in the product system and packaging [3,4].

The stability of PBBs in terms of viscosity and water-holding capacity (WHC) is significantly influenced by the plant source, storage temperature, and solid-to-water ratio [5]. This is closely associated with the protein, carbohydrate, lipid, and fiber content of the raw material, which in turn affects the overall stability of the final product. In addition, pretreatment, such as the roasting, soaking, and grinding of plant-based sources, influences the stability of PBBs during storage [4,5]. Food additives (emulsifiers and stabilizers) can improve the stability of PBBs (stable oil emulsion in water); however, these stabilizers are used in substantial amounts, which can positively affect the physical properties of the final product [6]. Furthermore, they can negatively impact consumers’ purchasing decisions because they are generally not 100% of their natural origin [7]. An alternative to improve the physiochemical properties of PBBs is the combination of different natural raw materials. This approach can enhance WHC and viscosity, particularly during storage [8]. Some alternatives include pea and rice mixes, resulting in a viscosity of ~0.15 Pa.s compared with rice-based beverages, with a viscosity of approximately 0.005 Pa.s over 143 days at 4 °C [9]. Other studies reported that the mix of legumes, such as lupin and chickpea, resulted in a viscosity of 2.15 Pa.s in comparison with non-mixed plant-based sources, such as only chickpeas (2.07 Pa.s), an oat-based beverage (1.85 Pa.s), and a hazelnut beverage (1.9 Pa.s) during 7 days at 4 °C, respectively [10]. These mixes improved the viscosity and physical properties compared with non-mixed PBBs, likely due to the interaction among the ingredients, such as protein, starch, and carbohydrates from different plant-based sources, under refrigerated conditions [5,9,10,11].

The microbiological quality is also a challenge in PBBs during storage [7]. Bacterial and fungal autochthonous in plant sources are difficult to eliminate using different processing technologies, such as soaking, roasting, and pasteurizing, because the microorganisms exhibit an increased heat tolerance at lower water activities induced by these processes [12]. Researchers have investigated different alternatives, including ultra-high temperature (UHT), to eliminate spoilage and foodborne pathogens and microorganisms in PBBs. However, the heat treatments used to reduce the microbial load may negatively affect the chemical and physical properties of the final PBBs and harm the nutritional composition of the food, due to the protein and starch modifications mainly related to its solubility and gelatinization [12,13,14].

Among the plant-based sources for preparing PBBs, quinoa (*Chenopodium quinoa* Willd.) has garnered significant attention in the recent decades. This is due to its high nutritional value, including gluten-free protein, vitamins, minerals, dietary fiber, and unsaturated fatty acids [15]. Quinoa proteins have a balanced profile of essential amino acids compared with cereals and pumpkin seeds [16]. In addition, quinoa has a unique flavor, and its combination with other plant sources, such as soybean, improves the physical and sensory properties of the prepared PBBs because of the high starch content (30–70%) in quinoa, which has good pasting properties and a high peak viscosity [8,17]. Another promising candidate for preparing PBBs is chickpea (*Cicer arietinum* L.), a legume widely consumed worldwide with a high protein content (23–29%) [18]. The technological properties of chickpeas include a high protein solubility, high WHC, and emulsification [19]. These properties can positively affect the stability of PBBs during storage, as reported by Aguilar et al. (2019) [20], where the syneresis of chickpea-based beverages was approximately 54.6–56.4% during 15 days of storage.

An interesting alternative for improving the stability and microbiological quality of PBBs during storage is fermentation with lactic acid bacteria (LAB). Fermentation erodes the starch granules, making their surfaces more porous, hydrolyzing part of the amorphous region, and reducing the molecular weight of the starch molecules, which could alter the functional attributes of food and improve its physical properties [21]. LAB can metabolize carbon sources, such as glucose and fructose, and transform them into lactic acid. Microbiological acidification, which is related to a high acidity, low pH, lactic acid, and bacteriocin production, can affect the presence of microbial contaminants and improve the safety of PBBs [7]. In addition, when probiotic bacteria are used in fermentation, this results in probiotic foods that contain a population of favorable microorganisms that benefit the host health when administered orally, generally at a dose of 10^6^–10^9^ CFU/mL(g) per day [22]. The main probiotic genus in food matrices is *Lactobacillus* spp., a strain that is widely used in industry to obtain high-quality fermentation products [23].

Some studies have reported that *Lactobacillus* spp. can grow in different plant-based matrices, and that the fermentation process can enrich the functional aspects of the finished products, thereby improving the physical stability and microbiological quality of PBBs throughout the storage period [24]. In addition, changes in the rheological properties have been reported during the processing of fermented cereal beverages stored at 4–6 °C for 21 days using a mixture of microorganisms, such as *Lactiplantibacillus plantarum, Lactobacillus casei, Pediococcus parvulus,* and *Saccharomyces cerevisiae*. These changes could be related to the increase in the bacterial and yeast cell concentrations in the sample, because storage under refrigeration conditions does not stop LAB growth [25,26].

Lactic acid fermentation by potential probiotic strains can affect the microbiological quality of PBBs during storage. The production of different organic acids (acetic and lactic acids) strongly affects the product quality in terms of pH, acidity, and syneresis [25]. During homolactic fermentation, lactic acid is the primary organic metabolite. This natural preservative enhances the preservation of PBBs during storage [27]. Ani et al. (2018) [28] reported that the microbiological quality of a fermented plant-based beverage produced with Bambara nut, soybean, and moringa seeds for 14 days showed a total decrease in coliforms and mesophilic aerobics. A low pH and high acidity levels related to lactic acid production by LAB acidify the environment, leading to the loss of viability or death of pathogenic microorganisms in PBBs. Furthermore, certain LAB can produce different bacteriocins that exert antimicrobial properties against pathogenic and food-spoilage microorganisms in PBBs [29]. *Lactobacillus acidophilus* LA-5 produces a bacteriocin called lactacin B, a peptide that exerts antimicrobial effects against pathogens, such as *Escherichia coli*, *Enterococcus* spp., *Clostridium* spp., *Cryptococcus* spp., and *Listeria* spp. [30]. Another factor that could be attributed to the improved microbiological quality is the low refrigeration temperature (4–10 °C), which limits the growth of mesophilic aerobic bacteria and coliforms [31].

The study of quinoa and chickpeas as plant-based sources for food fermentation has been explored separately (either quinoa or chickpeas alone) [8,10]. Because of the physical properties and protein quality of these raw materials, their combination can result in fermented beverages with an enhanced stability. Therefore, considering that quinoa flour provides a high water-holding capacity and good pasting properties, and chickpea flour offers a high protein solubility and emulsification properties, it is important to determine the stability of these combinations in fermented beverages during storage. Furthermore, it is important to understand the impact of different ratios of plant-based sources (e.g., the quinoa-to-chickpea ratio) on the physical and rheological properties of fermented PBBs. Therefore, this study aimed to evaluate the effects of the quinoa-to-chickpea flour ratio on the stability and physicochemical properties of fermented plant-based beverages during long-term storage. Specifically, this study aimed to assess changes in pH, acidity, viscosity, and microbial viability over a 50-day storage period at 8 °C to determine the potential of these fermented blends as stable food products.

## 2. Materials and Methods

### 2.1. Plant-Based Beverages Preparation

Quinoa seeds (*Chenopodium quinoa Willd*.) were obtained from Quinoa Fundo San Jose de Cáhuil (Cáhuil, O’Higgins Region, Chile) at the coordinates (34°28′44″ S 72°00′14″ W). Chickpea grains (*Cicer arietinum* L.) were purchased from a local supermarket (Santiago, Metropolitan Region, Chile) at the coordinates (33°26′29.4″ S, 70°42′45.6″ W). Quinoa seeds were washed with deionized water and dried for 24 h at 40 °C. Subsequently, the dried quinoa seeds and chickpea grains were milled using a laboratory-scale cross-beater mill–stainless steel grinding insert (Pulverisette 16, Fritsch, Idar-Oberstein, Germany) and sifted through a <250 μm sieve. The resulting flour was packed in airtight bags and stored at 4 ± 1 °C until further use. Freeze-dried probiotic cultures of *Lactobacillus acidophilus* LA-5 (Chr. Hansen, Hørsholm, Denmark) were grown in a de Man, Rogosa, and Sharpe (MRS) broth (Condolab, Madrid, Spain). The MRS broth was incubated at 38 ± 2 °C under anaerobic conditions for 24 h on an orbital shaker (SI500; Richmond Scientific, Chorley, UK). A sample (1 mL) of MRS broth was cultured in MRS agar at 38 ± 2 °C for 24 h to isolate the LAB. The activated culture was used for further inoculation. Quinoa flour (QF) and chickpea flour (CF) were mixed in a solid-to-water ratio of 1:7 (weight: volume). Three different proportions of QF and CF were used to produce the beverages: quinoa flour (90%) was mixed with 10% chickpea flour (QF90-CF10), 75% quinoa flour with 25% chickpea flour (QF75-CF25), and 50% quinoa flour with 50% chickpea flour (QF50-CF50) (weight: weight). The beverages were pasteurized in a water bath WNB 14 (Memmert, Schwabach, Germany) at 75 °C for 15 min (to decrease the autochthonous microorganisms present in the raw material) and homogenized at 14,000 revolutions per minute (rpm) with an OV5 homogenizer (VELP Scientific, Inc., Suffolk, NY, USA). *Lactobacillus acidophilus* LA-5 was inoculated into the prepared beverages at a concentration of 10% (*w*/*v*), and the mixtures were fermented at 38 ± 1 °C for 10 h until the pH of each beverage reached less than 4.5 units. The resulting plant-based fermented beverages were stored for 50 days at 8 ± 1 °C in the dark. During storage, samples were collected every 10 days to study the physicochemical stability and microbiological quality.

### 2.2. Chemical Analysis during Storage

#### Determination of pH, Titratable Acidity, Water-Soluble Solids, and Organic Acids

The pH of each sample was measured using a pH meter (HI5521; Hanna Instruments, Smithfield, RI, USA). For the titratable acidity (TTA) determination, 5 mL of the sample was diluted in 45 mL of deionized water and titrated with 0.1N NaOH solution until a pH of 8.3–8.5 was achieved, using phenolphthalein (0.1% *w*/*v* in 95% ethanol) as an indicator. TTA, expressed as an acid constant, was calculated as follows:

(1) $$TTA\;\left(\%\right)=\frac{Volume\;of\;NaOH\;\left(\mathrm{mL}\right)\times Normality\;of\;NaOH\times 0.090}{Volume\;of\;the\;sample\;\left(\mathrm{mL}\right)}\times 100$$

The water-soluble solids were evaluated using three drops of the homogenized sample and measured using a refractometer (HI96800; Hanna Instruments, Smithfield, RI, USA). The water-soluble solids were expressed in Brix degrees. The organic acids (lactic acid, acetic acid, and ascorbic acid) were measured using the photometer analyzer Y15 (Biosystems, Barcelona, Spain), according to the manufacturer’s instructions. Samples (1 mL) of each beverage were centrifuged (15,000 rpm × 5 min, 8 °C), and 500 μL of the supernatant was used for the measurements. 

### 2.3. Physical Analysis during Storage

#### Determination of the Water-Holding Capacity

The water-holding capacity (WHC) of the plant-based beverages was analyzed according to Xu et al. (2022) [32]. In total, 50 mL of each sample were weighed and placed in conical sterile polypropylene centrifuge tubes. The tubes were centrifuged at 5000 rpm for 30 min at 4 ± 2 °C in a Hettich centrifuge (Universal 320 model/Andreas Hettich GmbH & Co. KG, Föhrenstr. 12, D–78532, Tuttlingen, Germany). After centrifugation, the supernatant was carefully removed, and the resulting gel was accurately weighed. The WHC was calculated using the following formula:

(2) $$WHC\;\left(\%\right)=\frac{m2}{m1}\times 100$$

where m1 is the total weight of the sample and m2 represents the gel formation mass after the sample centrifugation, respectively. All the samples were measured during storage. 

### 2.4. Rheological Measurements during Storage

The rheological stability of the samples was measured using a rheometer (Discovery HR2, TA Instruments, New Castle, DE, USA) equipped with a flat parallel plate geometry (stainless steel, 50 mm diameter, 1000 µm gap) following the method described by Quilaqueo et al. (2022) [33] with some modifications. The samples were carefully placed on a plate and covered with a solvent trap to maintain the temperature. The TRIOS software package (TA Instruments, New Castle, DE, USA) was used to control the equipment and acquire the rheological parameters. The steady-shear flow measurements were taken at 8 °C in a 0.1–150 s^−1^ shear rate range. The range of linear viscosity values for the samples was obtained from the plot of the elastic modulus (G′) vs. the oscillatory strain (%). In addition, the viscoelastic behavior of the samples was measured under oscillatory conditions at 1 Hz and from 0.01 to 20%. In the frequency sweep test, the temperature was maintained at 8 °C, and the response of the moduli (G′, G″) to the increasing frequency (0.1 to 100 Hz) at a strain of 0.2% within the linear viscosity was measured. All the samples were analyzed during the storage period. Three replicates of each sample were recorded for each test.

### 2.5. Microbiological Quality

Bacterial and fungal counts were assessed to determine the microbial quality (presence of bacteria and fungi) of the PBBs during storage. Aliquots (10 mL) of the plant-based beverages were retrieved from each fermented flask after homogenization for 50 days at 8 °C. Each sample was serially diluted in 90 mL sterile 1% (*w*/*v*) peptone water. Five serial dilutions were performed, and each microorganism was counted in the three most appropriate dilutions. Aerobic mesophiles, total coliforms, fungi, and yeast were enriched using trypto-casein soy agar (TSA), Violet Red Bile Glucose agar (VRBG), and Yeast Glucose Chloramphenicol (YGC) agar (Condolab, Madrid, Spain), respectively. The inoculated plates were incubated at 37 ± 2 °C for 24 h to determine the bacterial counts, whereas the plates for the fungal counts were incubated at 25 ± 1 °C for 72 h. The results were expressed as CFU/mL.

### 2.6. Probiotic Viability 

LAB viability during storage was determined using MRS agar. Briefly, the beverage samples were serially diluted (1:10 *v*/*v*) in buffered peptone water (1% *w*/*v*) (Condolab, Madrid, Spain). The diluted samples were spread on MRS agar and incubated for 72 h at 38 ± 2 °C under anaerobic conditions. MRS plates containing 25–250 LAB colonies were used to determine the LAB concentration. The results were expressed as CFU/mL. 

### 2.7. Statistical Analysis 

All treatments and analyses were in triplicate (n = 3). The results are reported as mean ± standard deviation. The experimental data were tested using the Shapiro–Wilk test to assess normality. The statistical significance was tested using a one-way analysis of variance (ANOVA) at a significance level of *p* < 0.05. The differences between the sample sets were resolved using the LSD method at 95% confidence and the Statgraphics Centurion XVI.I software (Manugistics, Inc., Rockville, MD, USA) for Windows.

## 3. Results and Discussion

### 3.1. Chemical Properties of Fermented Plant-Based Beverages during Storage

**Figure 1:**
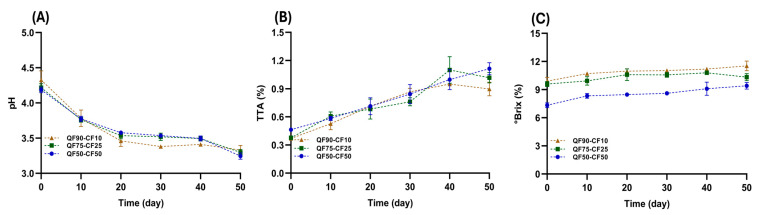
Changes in (**A**) pH, (**B**) TTA concentration, and (**C**) Brix degrees of beverages prepared with different ratios of quinoa flour (QF) and chickpea flour (CF) for 50 days at 8 °C. Quinoa flour (90%) was mixed with 10% chickpea flour (QF90-CF10) brown, 75% quinoa flour with 25% chickpea flour (QF75-CF25) green, or 50% quinoa flour with 50% chickpea flour (QF50-CF50) blue. Data are shown as the mean ± SD (n = 3).

The pH and acidity of fermented PBBs are important parameters that determine the product quality during storage because of their chemical stability. Figure 1 shows the pH, TTA values, and Brix degrees and Table 1 shows the organic acid production observed during the 50-day storage period under refrigerated conditions. A significant decrease in pH was observed after approximately 10 days of storage, which increased the TTA, probably due to lactic acid production from the metabolism of *Lactobacillus acidophilus*. During storage for 10–30 days, the pH values of the fermented beverages were stable, ranging from 3.7 to 3.5 across all the samples. At the end of the storage period, the pH value of each PBB was approximately 3.3 (Figure 1A), suggesting that *Lactobacillus acidophilus* can resist refrigeration temperatures and metabolize carbon sources during long storage periods (50 days) [8]. Additionally, the TTA values were consistently between 0.6% and 0.75%, which correlated with the pH values observed over a short period (0–30 days), indicating the high chemical stability of the fermented beverages during this time. In addition, there was an increase in organic acids over a short and long storage period. However, at the end of the long storage period, no significant differences were observed in the pH values of the blends, although QF50-CF50 presented the highest TTA among all the mixtures (Figure 1B and Table 1). This post-acidification by lactic acid is an undesirable process in PBBs, which can cause acidity, shorten the shelf life, and lead to syneresis [8]. Therefore, for further studies, it is recommended to extend the initial fermentation time to more than 10 h, until no significant differences in pH and acidity are observed.

In addition, no effect of different quinoa-to-chickpea ratios on the pH of the samples was observed after 50 days of storage. The lowest TTA values and organic acid production were observed for the QF90-CF10 (0.8%; 4.3 g/L) blend, followed by QF75-CF25 (1.0%; 4.7 g/L), and the QF50-CF50 (1.1%; 5.6 g/L) blend had the highest TTA (Figure 1B and Table 1) at the end of storage. This effect is directly related to the increase in CF in PBBs. According to Liu et al. (2023) [34], chickpeas have a higher protein content than quinoa. This increase in protein content may influence the TTA and organic acid values owing to the better buffering capacity of CF, and more lactic acid production by *Lactobacillus acidophilus* could be required to reduce the pH of the beverages during the storage period. A similar result was reported by Huang et al. (2022) [8] for the PBBs prepared using different proportions of quinoa and soybeans. The TTA of the 60% quinoa–40% soybean blend was approximately 0.3% compared with that of 100% soybean, with a TTA value of 0.73, indicating that the presence of a legume (soybean) affected the TTA of the plant-based fermented beverage at the end of the storage period. 

Furthermore, the different ratios of QF and CF affected the Brix degrees at the end of the 50 days of storage. The values were approximately 11.5%, 10.3%, and 9.4% for QF90-CF10, QF75-CF25, and QF50-CF50, respectively, indicating that increased CF decreased the water-soluble solids (mainly sucrose and raffinose) in the PBBs. According to Rincon et al. (2020) [35], chickpea-based beverages have a water-soluble solid content of approximately 4.04 Brix degrees. Meanwhile, as reported by Cerdá-Bernad (2021), quinoa-based beverages contain water-soluble solids of approximately 6.25 Brix degrees [36]. Our results agree with those of Huang et al. (2022) [8], who reported that a high soybean flour content decreased water-soluble solids in a PBB blend with quinoa. It could be clarified why the CF content decreased the Brix degree in the fermented PBBs. 

In contrast, the Brix degrees did not show any differences in any of the fermented PBBs at the end of the storage period (50 days) (Figure 1C). This could be explained by the preference for glucose and fructose (readily fermentable monosaccharides) by *Lactobacillus* strains over other soluble oligosaccharide sources, such as maltose, sucrose, and raffinose, by *Lactobacillus acidophilus* during storage [37]. These carbon sources (glucose and fructose) were likely sufficient to sustain the bacterial activity throughout the storage period. To the best of our knowledge, there is no information available on the stability of fermented beverages made with quinoa and chickpeas, but similar changes have been reported for coconut milk fermented beverages, although these studies evaluated shorter storage periods (0–30 days) [37]. 

### 3.2. Physical Stability of Fermented Plant-Based Beverages during Storage

**Figure 2:**
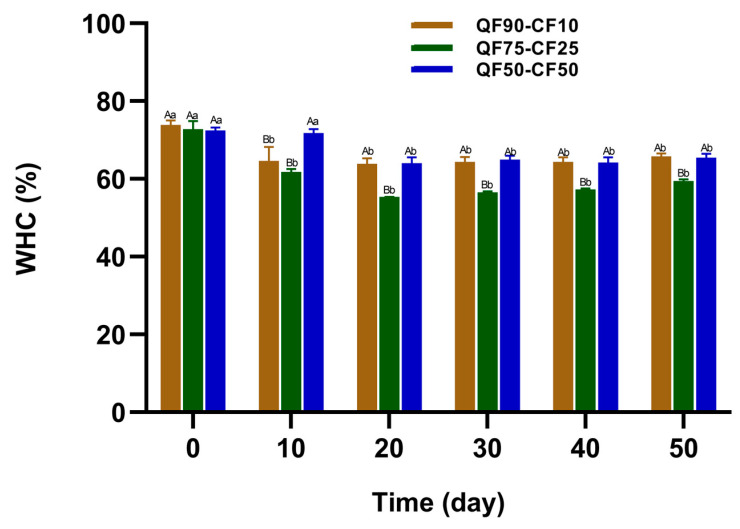
WHC (%) of fermented plant-based beverages during storage for 50 days at 8 °C. Quinoa flour (90%) was mixed with 10% chickpea flour (QF90-CF10) brown, 75% quinoa flour with 25% chickpea flour (QF75-CF25) green, or 50% quinoa flour with 50% chickpea flour (QF50-CF50) blue. Data are shown as the mean ± SD (n = 3). Means with different lowercase letters (a–c) indicate significant differences (*p* < 0.05) between the different ratios of QF–CF on the same day of storage. Means with different uppercase letters (A–C) indicate significant differences (*p* < 0.05) between the same ratio of QF–CF at different storage times.

The physical stability of different PBBs over 50 days at 8 °C was studied and expressed according to the water-holding capacity (WHC, %) of each beverage, as shown in Figure 2. WHC is an essential characteristic of PBBs because it can affect the sensory acceptance and limit the shelf life [8]. A significant decrease in WHC was observed after approximately 10 days of storage, which remained stable until 50 days under storage conditions. At the beginning of storage, the blend with QF90-CF10 had a WHC of approximately 75% with a low liquid separation. However, the higher CF content of the QF75-CF25 and QF50-CF50 blends resulted in WHC values of 71% and 69%, respectively. These results suggest that a high CF content adversely affects this physical property. This could be related to the lower starch content (28.4–31.2%) in chickpeas [38], whereas the starch content of quinoa is 30–70% [17]. At the end of storage (50 days), WHC was stable at approximately 65% in all samples. Syneresis is a PBB defect that occurs during storage and is related to liquid expelled from a gel-like structure [17]. A lower WHC may result in increased syneresis. The factors that can affect the syneresis of fermented PBBs are low WHC, storage temperature, pretreatment of the plant source, and the starter culture used in the fermentation process [39]. Furthermore, according to Checkdid et al. (2021), fermented plant-based products containing starch may undergo retrogradation during cooling, causing water to be expelled from the gel and resulting in syneresis [40]. Similarly, it has been reported that the protein concentration, pH, and temperature affect WHC in PBBs during storage [4]. In addition, variations in the lower WHC values in quinoa–chickpea-based fermented beverages (Figure 2) could be associated with a higher content of soluble solids in the system, which can increase with storage time [41]. In turn, changes in WHC may be associated with the varying ability of lactic acid bacteria to hydrolyze different protein subunits and gelatinized starch granules, influencing the particle size distribution, zeta potential, and intermolecular forces in fermented PBBs during storage [42]. Likewise, it has been reported that quinoa proteins have a higher WHC than legume proteins, such as soybean; thus, in various food applications, quinoa is expected to improve the physical characteristics [43]. Our results agree with those of Huang et al. (2022) [8], who reported a WHC of approximately 100% for quinoa-based beverages. However, when the ratio of soybeans increased, the WHC was lower (approximately 57.6%) and decreased over 21 days under storage conditions (4 °C). 

### 3.3. Changes in the Rheological Properties of Fermented Plant-Based Beverages during Storage

**Figure 3:**
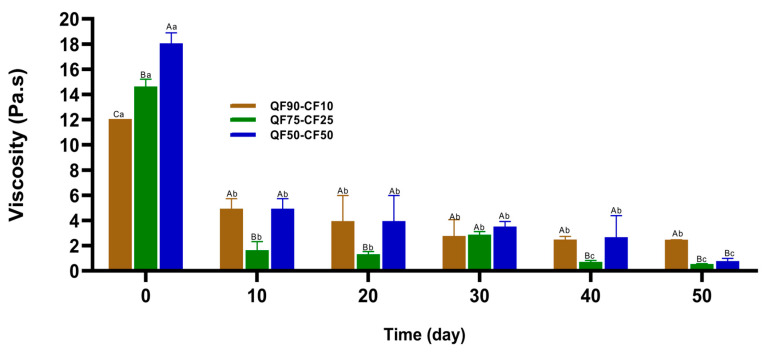
Changes in the viscosity of fermented plant-based beverages during storage for 50 days at 8 °C. Quinoa flour (90%) was mixed with 10% chickpea flour (QF90-CF10) brown, 75% quinoa flour with 25% chickpea flour (QF75-CF25) green, or 50% quinoa flour with 50% chickpea flour (QF50-CF50) blue. Data are shown as the mean ± SD (n = 3). Means with different lowercase letters (a–c) indicate significant differences (*p* < 0.05) between the different ratios of QF–CF on the same day of storage. Means with different uppercase letters (A–C) indicate significant differences (*p* < 0.05) between the same ratio of QF–CF at different storage times.

**Figure 4:**
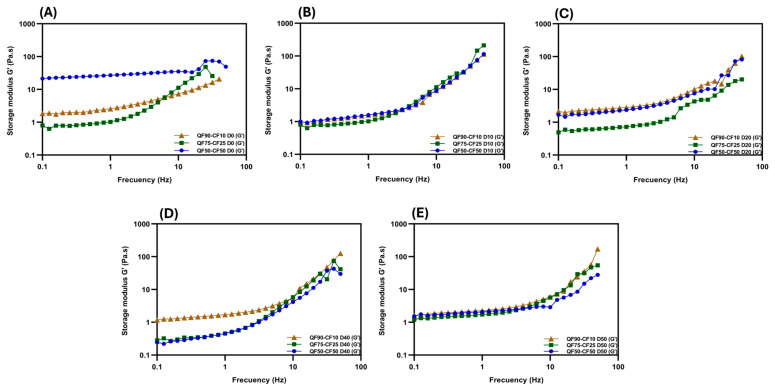
Changes in viscoelasticity (storage modulus, G′) of fermented plant-based beverages at 0 (**A**), 10 (**B**), 20 (**C**), 40 (**D**), and 50 (**E**) days of storage at 8 °C. Quinoa flour (90%) was mixed with 10% chickpea flour (QF90-CF10) brown, 75% quinoa flour with 25% chickpea flour (QF75-CF25) green, or 50% quinoa flour with 50% chickpea flour (QF50-CF50) blue. Data are shown as the mean ± SD (n = 3).

The rheological properties of the fermented beverages with three different quinoa-to-chickpea ratios during storage were evaluated, and the results are presented in Figure 3 and Figure 4. The viscosity of the flour-based beverages depends on the starch content (including the type of starch and retrogradation) and the type of protein [44]. At the beginning of storage, the blend with QF90-CF10 had a lower viscosity of approximately 12.05 Pa.s, followed by QF75-CF25, with a viscosity of 14.60 Pa.s, and QF50-CF50 exhibited a higher viscosity of 18.05 Pa.s (Figure 3). This suggests that a high CF content positively affected the viscosity of the fermented beverages at the beginning of the storage period (0–30 days). It has been reported that starches with a high amylopectin content can absorb and retain more water, and thus show a higher viscosity [45,46,47]. Additionally, after the fermentation process, the molecular weight of starch can decrease because of the exo-amylases secreted by LAB [26,44,45]. Furthermore, the fermentation process causes a significant change in the amylose-to-amylopectin ratio; thus, the amylose content can increase, which can favor starch retrogradation [26]. Some studies have reported that viscosity can decrease with high amounts of quinoa flour, which is related to the higher firmness of the fermented beverages because of the increased restriction of molecular motion due to entanglements between the polymer chains [48]. In addition, at the end of lactic acid fermentation, the protein content on the surface of the starch granules could be reduced, which could enhance the swelling of the starch granules and increase the viscosity of the PBBs with higher amounts of CF [26,47]. In our study, chickpeas had a higher protein content than quinoa, and an acidic environment (pH 4.3) could induce the gelation of proteins. This phenomenon may create a network that traps water and other components, thereby increasing the viscosity of PBB [8]. Another factor that could explain the higher viscosity in PBBs with more CF is the high protein solubility of QF75-CF25 and QF50-CF50 after fermentation and at the beginning of storage (Table S1). This could be related to structural changes in the protein during fermentation, which might increase the hydrophilic sites, allowing for more protein–water interactions, thereby increasing the viscosity [45]. Interestingly, a significant decrease in viscosity (*p* < 0.05) was observed after 50 days of storage. Some studies have reported that this effect can be explained by the rapid depolymerization of amylopectin and the release of more linear chains, or by an increase in the chain length through the formation of intramolecular and intermolecular linkages between amylose residues, impacting the viscosity [26]. Additionally, it has been reported that the viscosity of fermented beverages can decrease at the end of storage, possibly related to long-term starch retrogradation [49]. Furthermore, post-acidification could result in a loose protein network structure, which might unbalance protein–protein interactions and impact the viscosity at the end of the storage time [26,42].

Conversely, after 50 days of storage, the blend that presented the highest viscosity was QF90-CF10 (2.47 Pa.s), followed by QF50-CF50 (0.78 Pa.s) and QF75-CF25 (0.54 Pa.s). These results indicate that the high CF content in PBBs could have the opposite effect on viscosity during long storage periods (50 days). Likewise, quinoa starch granules are characterized by excellent stability during retrogradation and freezing [48,49,50,51]. Therefore, the duration of starch retrogradation could promote a decrease in hydrogen bonds and other interactions with peptides, thus decreasing the viscosity of the beverages [48]. Similarly, chickpea starch can offer a weaker network structure than quinoa starch, which is related to the starch content and retrogradation of these plant-based sources [8,17]. In addition, the hydrolysis of proteins by bacterial enzymes produced by *Lactobacillus acidophilus* during storage could reduce the molecular size of the proteins and peptide interactions and decrease the viscosity of the PBBs at the end of the storage period [26]. Chen et al. (2018) [50] reported a decrease in the viscosity of beverages with a high content of CF during storage related to the high activity of microorganisms, which influenced the protein-network interaction and, consequently, the product viscosity. This could explain why PBBs with a higher CF content presented lower viscosities at the end of the storage period. 

Figure 4 shows the changes in the viscoelasticity of the PBBs during storage. All the plant-based beverages exhibited weak viscoelastic gel behavior (G′ > G″). No crossover was observed between G′ and G″, indicating a strong solid-like structure (Figure 4 and Figure S1). Differences in the G’ values were detected throughout storage for both the short and long storage periods. At the beginning of storage (0 days), the QF90-CF10 and QF75-CF25 blends had the lowest G’ values. Meanwhile, QF50-CF50 presented the highest G′, indicating that the CF content could positively impact the G′ of the PBBs. The high G′ phenomenon in a short time could be mainly attributed to the increased chickpea protein and chickpea starch content, which could be related to the strengthening of the protein–protein complexes and chickpea starch with higher amounts of amylose content and long-branch chain amylopectin, affecting the retrogradation patterns, probably resulting in a higher G′ value [33,51]. After 40 days of storage, the presence of CF affected G′, where QF75-CF25 and QF50-CF50 presented the lowest G′ and QF90-CF10 presented the highest G′, showing that the effect was reversed. Furthermore, quinoa starch tends to retrograde slowly, mainly because of its low amylose content, and the high content of soluble fiber seems to retard retrogradation [52]. In addition, the high protein content of CF can decrease the G′ values during refrigerated starch retrogradation [51,52,53]. This may explain why more CF had a negative impact on the viscosity and viscoelasticity during long-term storage.

### 3.4. Microbiological Quality during Storage

The microbiological quality of the different fermented PBBs during 50 days of storage is shown in Table 2. The microbial concentrations observed in the fermented plant-based beverages during storage and the QF-to-CF ratios were not significantly different. At the beginning of the storage period (day 0), aerobic mesophiles and coliforms were detected. Mesophilic bacteria remained on day 10. However, the microbial load decreased by approximately 99% by the end of the storage period. Coliforms were not detected at this time. This may be attributed to the fact that fermentation can acidify PBBs due to its low pH, which limits the increase in pathogens. In addition, LAB can produce different metabolites and peptides (lactic acid and bacteriocins) that have antimicrobial activity on prepared PBBs [30]. Another factor that can be attributed to the microbiological quality of PBBs is the low refrigeration temperature (8 °C), which limits the growth of pathogenic microorganisms. Ani et al. (2018) [28] reported a decrease in the aerobic mesophile count and the absence of coliforms during the storage time (14 days), related to the highly acidic environment in the PBBs based on soybean, Bambara nut, and moringa oleifera seeds. In addition, according to the Colombian Ministry of Health and Social Protection, in standard (1407/2022), the PBBs prepared are within the established microbiological limits, making them safe for human consumption [54].

### 3.5. Probiotic Viability under Storage Conditions

**Figure 5:**
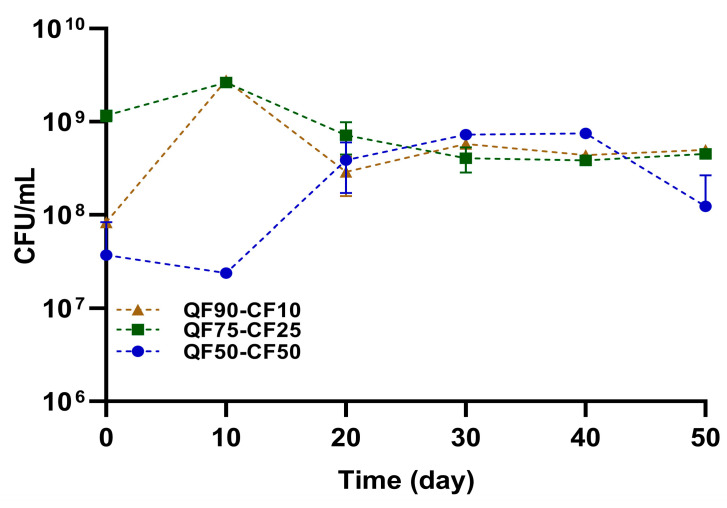
LAB viability in plant-based beverages during storage for 50 days at 8 °C. Quinoa flour (90%) was mixed with 10% chickpea flour (QF90-CF10) brown, 75% quinoa flour with 25% chickpea flour (QF75-CF25) green, and 50% quinoa flour with 50% chickpea flour (QF50-CF50) blue. Data are shown as the mean ± SD (n = 3).

The viability of LAB during storage is shown in Figure 5. The viability of LAB in PBBs was relatively stable during the 50-day storage period for all the blends. The highest LAB count was observed in PBB with QF90-CF10, followed by QF75-CF25 at approximately 1 × 10^9^ CFU/mL, and QF50-CF50 had the lowest LAB count of approximately 1 × 10^8^ CFU/mL at the end of the storage period. Between 0 and 10 days of storage, there was a slight decrease in LAB in the QF50-CF50 blend. This could be related to the abundance of nutrients at the beginning of the storage period, which causes competition for nutrients by starter, and other microbes or autochthonous LAB [55]. However, after some time, the probiotics reached a stationary phase. During the 30 days of storage, no significant differences were observed between the beverages with different quinoa-to-chickpea ratios, indicating that the increase in CF did not affect the viability of the probiotic. At the end of the storage period (50 days), QF50-CF50 exhibited the lowest viability of the starter culture. This could be attributed mainly to the high acidity stress in the PBBs with a higher proportion of CF, which inhibits LAB growth by acidifying the environment according to the pH and TTA values reported in Figure 1. Similar results were reported by Sidhu et al. (2020) [22], where LAB were less tolerant to PBBs fortified with 5% chickpea flour. In addition, previous studies have reported that *Lactobacillus acidophilus* can survive better under acidic conditions [56]. This is related to the ability of LAB to resist changes in the cytoplasmic buffering capacity and membrane H^+^ conductance [19,22]. Although the increase in acidity was related to the high CF content, LAB maintained a satisfactory viability (>1 × 10^6^ CFU/mL) in all PBBs under storage conditions, indicating the probiotic character of PBBs [19,20,21].

## 4. Conclusions

This study demonstrated the impact of the quinoa-to-chickpea ratio on the physicochemical properties and microbiological quality of fermented plant-based beverages (PBBs) during long-term storage. The increase in LAB colonies during storage produced a low pH and high acidity, suggesting that the quinoa-to-chickpea ratios studied were suitable substrates for *Lactobacillus acidophilus* growth and maintenance during short- and long-term refrigerated storage. The microbiological quality remained consistent over the shelf life of the PBBs. The incorporation of chickpea flour into PBBs positively affected physical properties, such as WHC, viscosity, and viscoelasticity, between 0 and 30 days of storage. The results showed that 50% quinoa and chickpea flours resulted in a higher viscosity and LAB counts during the short-term storage time. This indicates that a higher proportion of chickpea flour enhanced these parameters during this period, possibly because chickpea starch contains higher amounts of amylose content and long-branch chain amylopectin, which impacted the retrogradation pattern of the fermented starches. Conversely, the same quinoa-to-chickpea ratio (50%) resulted in a higher acidity, lower viscosity, and lower LAB count after 50 days of storage, indicating that the presence of chickpea flour had the opposite effect on these parameters under prolonged refrigerated conditions. The 90% quinoa and 10% chickpea flour ratios presented a higher viscosity related to the good pasting properties of the quinoa starch and its retrogradation pattern during long-term storage. These results can help to improve the understanding of the effects of different ratios of plant-based sources on physicochemical properties during extended storage.

## Figures and Tables

**Table 1:** Production of organic acids (lactic, acetic, and ascorbic acid) during storage time.

Composition		QF90-CF10	QF75-CF25	QF50-CF50
Time (Day)	Organic Acid (g/L)			
0	Lactic acid	1.9 ± 0.25 ^b^	2.1 ± 0.17 ^b^	2.6 ± 0.15 ^a^
	Acetic acid	0.0 ± 0.0 ^a^	0.0 ± 0.0 ^a^	0.0 ± 0.0 ^a^
	Ascorbic acid	BDL	BDL	BDL
30	Lactic acid	5.1± 0.01 ^a^	4.6 ± 0.04 ^b^	5.6 ± 0.02 ^a^
	Acetic acid	1.1 ± 0.2 ^a^	0.7 ± 0.4 ^b^	0.7 ± 0.4 ^b^
	Ascorbic acid	BDL	BDL	BDL
50	Lactic acid	4.3 ± 0.03 ^b^	4.7 ± 0.05 ^b^	5.6 ± 0.01 ^a^
	Acetic acid	1.1 ± 0.8 ^a^	0.6 ± 0.6 ^b^	0.7 ± 0.8 ^b^
	Ascorbic acid	BDL	BDL	BDL

Different superscript letters in columns for the same row indicate statistically significant differences (*p* < 0.05), BDL: Below detection limit. Data are shown as the mean ± SD (n = 3).

**Table 2:** Microbiological quality of plant-based beverages during the storage time.

Composition		QF90-CF10	QF75-CF25	QF50-CF50
Time (Day)	Cell Count (CFU/mL)			
0	AMC	5.4 × 10^1 a^	2.87 × 10^1 a^	3.01 × 10^1 a^
	Fungi	ND	ND	ND
	Coliforms	1 × 10^1 a^	ND	1 × 10^1 a^
10	AMC	1.3 × 10^1 a^	1.1 × 10^1 a^	1 × 10^1 a^
	Fungi	ND	ND	ND
	Coliforms	ND	ND	ND
20	AMC	ND	ND	ND
	Fungi	ND	ND	ND
	Coliforms	ND	ND	ND
30	AMC	ND	ND	ND
	Fungi	ND	ND	ND
	Coliforms	ND	ND	ND
40	AMC	ND	ND	ND
	Fungi	ND	ND	ND
	Coliforms	ND	ND	ND
50	AMC	ND	ND	ND
	Fungi	ND	ND	ND
	Coliforms	ND	ND	ND

Different superscript letters in columns for the same row indicate statistically significant differences, ND: not detected (no visible colony or less than 10 CFU/mL). AMC, aerobic mesophiles count; Fungi, yeast, and mold; Coliforms, total coliforms. Data are shown as the mean ± SD (n = 3).

## Data Availability

The original contributions presented in the study are included in the article/Supplementary Materials, further inquiries can be directed to the corresponding author.

## Supplementary Materials

The following supporting information can be downloaded at https://www.mdpi.com/article/10.3390/foods13152462/s1; Word file supplementary materials contain as follows: Figure S1. Changes in viscoelasticity (loss modulus, G″) of fermented plant-based beverages at 0 (A), 10 (B), 20 (C), 40 (D), and 50 (E) days of storage at 8 °C. Quinoa flour (90%) was mixed with 10% chickpea flour (QF90-CF10) brown, 75% quinoa flour with 25% chickpea flour (QF75-CF25) green, or 50% quinoa flour with 50% chickpea flour (QF50-CF50) blue. Data are shown as the mean ± SD (n = 3); Table S1. Total protein content and protein solubility of fermented plant-based beverages. Different superscript letters in columns for the same row indicate statistically significant differences (*p* < 0.05). Data are shown as the mean ± SD (n = 3).
