# Supplementary material for: Role of Quinoa (Chenopodium quinoa Willd) and Chickpea (Cicer arietinum L.) Ratio in Physicochemical Stability and Microbiological Quality of Fermented Plant-Based Beverages during Storage
Source: Foods. 2024 Aug 4;13(15):2462. doi: 10.3390/foods13152462 (PMC11312257; doi:10.3390/foods13152462)
Supplement: Supplementary file 1 [file foods-13-02462-s001.zip › foods-3127141-supplementary.pdf]

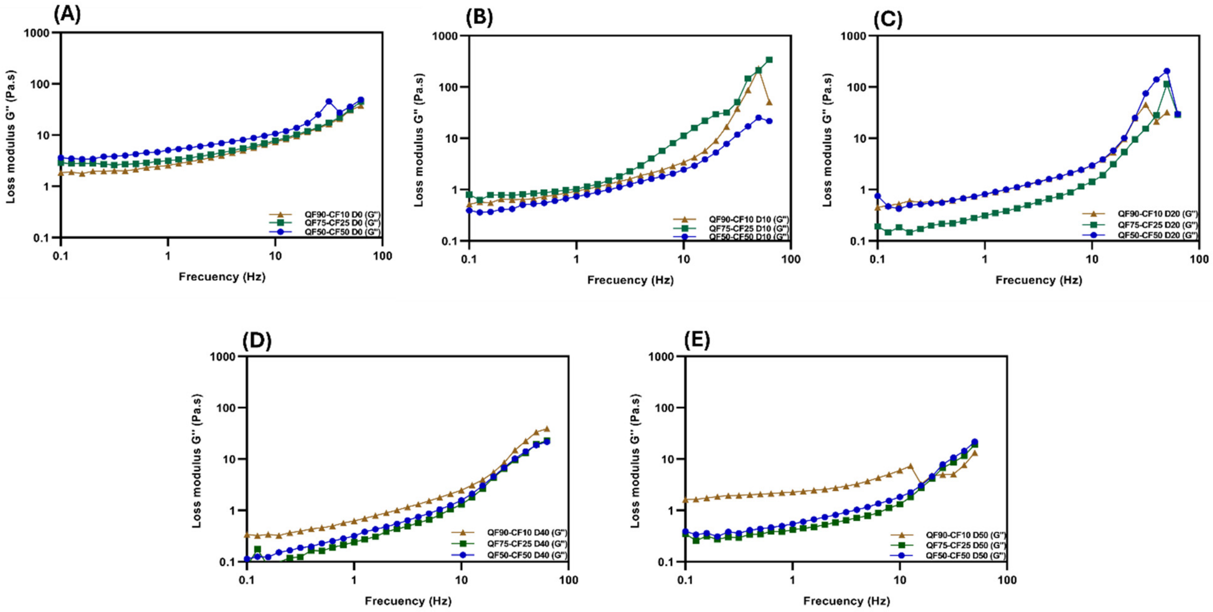

Figure S1: changes in viscoelasticity (loss modulus, G´´) of fermented plant-based beverages at 0 (**A**), 10 (**B**), 20 (**C**), 40 (**D**), and 50 (**E**) days of storage at 8 °C. Quinoa flour (90%) was mixed with 10% chickpea flour (QF90-CF10) brown, 75% quinoa flour with 25% chickpea flour (QF75-CF25) green, or 50% quinoa flour with 50% chickpea flour (QF50-CF50) blue. Data are shown as the mean ± (n = 3)

Table S1. Total protein content and protein solubility of fermented plant-based beverages

	Beverage		
**Parameters**	**QF90-CF10**	**QF75-CF25**	**QF50-CF50**
Total protein content (%)	1.6 ± 0.15[a]	1.9 ± 0.01[b]	2.3 ± 0.16[b]
Protein solubility (%)	32.1 ± 1.81[a]	35.1 ± 0.86[a]	43.6 ± 0.81[b]

Different superscript letters in columns for the same row indicate statistically significant differences ($p < 0.05$). Data are shown as the mean ± SD (n = 3)
